# Association Between Season of Birth and External Risk Factors in Patients With Hashimoto’s Thyroiditis and Non-autoimmune Hypothyroidism

**DOI:** 10.7759/cureus.114663

**Published:** 2026-08-17

**Authors:** Danijel Gajic, Emina Bajric Custo, Dzana Nuhanovic, Belkisa Izic, Branislav Milic, Darko Gajic, Senada Selmanovic, Sabina Ademovic Dzambic, Sanda Kreitmayer Pestic

**Affiliations:** 1 Department of Family Medicine, Health Center Orašje, Orašje, BIH; 2 Department of Family Medicine, Health Center Lukavac, Lukavac, BIH; 3 Clinic for Radiology and Nuclear Medicine, Department of Nuclear Medicine, University Clinical Center Tuzla, Tuzla, BIH; 4 Department of Psychiatry, Health Center Orašje, Orašje, BIH; 5 Department of Microbiology, Public Health Center Orašje, Orašje, BIH; 6 Department of General/Family Medicine, Center for Education and Professional Training, Public Health Institution Health Center “Dr. Mustafa Šehović” Tuzla, Tuzla, BIH; 7 Faculty of Medicine, Department of Family Medicine, University of Tuzla, Tuzla, BIH; 8 Department of General/Family Medicine, Public Health Institution Health Center “Dr. Mustafa Šehović” Tuzla, Tuzla, BIH

**Keywords:** body mass index, hashimoto’s thyroiditis, hypothyroidism, season of birth, vitamin d

## Abstract

Background

Both Hashimoto’s thyroiditis and non-autoimmune hypothyroidism are highly prevalent thyroid disorders. While their clinical presentations overlap, they are driven by distinct pathophysiological mechanisms and potentially influenced by different genetic susceptibilities and environmental triggers, including prenatal solar exposure and vitamin D status. This study aimed to evaluate and compare the season of birth, serum vitamin D levels, body mass index (BMI), smoking habits, and family history of thyroid diseases between patients with Hashimoto’s thyroiditis and patients with non-autoimmune hypothyroidism in Tuzla Canton.

Materials and methods

This retrospective observational cross-sectional study reviewed the medical records of 200 patients evaluated and followed up at the Department of Nuclear Medicine and Radiology of a tertiary care university hospital in Bosnia and Herzegovina. Participants were equally divided into two groups: patients with Hashimoto’s thyroiditis (n = 100) and patients with non-autoimmune hypothyroidism (n = 100). Numerical variables were analyzed using the Mann-Whitney U and Kruskal-Wallis tests, while categorical variables were evaluated using the χ² test. Post hoc pairwise comparisons were performed with Bonferroni correction.

Results

The study population comprised 189 women (94.5%) and 11 men (5.5%). The distribution of birth seasons did not differ significantly between the groups (χ² = 2.444, df = 3, p = 0.485). Similarly, no statistically significant differences were observed in median serum vitamin D levels (p = 0.931) or smoking rates (23.0% in the Hashimoto’s group vs. 22.0% in the hypothyroid group, p = 0.866). However, patients with non-autoimmune hypothyroidism were significantly older (median: 59.0 vs. 49.0 years, p < 0.001) and presented with significantly higher BMI values (median: 28.20 kg/m² vs. 26.21 kg/m², p = 0.008) compared to those with Hashimoto’s thyroiditis. A positive family history of thyroid disease was significantly more frequent among patients with Hashimoto’s thyroiditis than those with hypothyroidism (62.0% vs. 47.0%, χ²= 4.537, df = 1, p = 0.033). Within the non-autoimmune hypothyroidism group, vitamin D levels differed significantly across BMI categories (p = 0.030), with overweight patients exhibiting significantly lower median vitamin D levels compared to normal-weight patients (median: 18.50 vs. 24.00, adjusted p = 0.045).

Conclusion

Season of birth, smoking status, and overall serum vitamin D levels do not distinguish Hashimoto’s thyroiditis from non-autoimmune hypothyroidism. However, patients with non-autoimmune hypothyroidism present with higher BMI, and those who are overweight are particularly prone to lower vitamin D levels. In contrast, Hashimoto’s thyroiditis is more strongly characterized by a positive family history of thyroid disease, highlighting a more pronounced hereditary component.

## Introduction

The thyroid gland is positioned in the anterior region of the neck and plays a major role in metabolic regulation through its hormones T3, T4, and calcitonin [1]. Disorders of thyroid function manifest as decreased or increased secretion of its hormones. Hypothyroidism is far more common, affecting between 0.2% and 5.3% of the global population, with a higher prevalence in females [2]. Depending on the primary etiology, hypothyroidism can be classified as primary (direct damage to the gland), secondary (adenohypophyseal dysfunction), or tertiary (hypothalamic dysfunction). Differentiation between secondary and tertiary forms in clinical practice is difficult, so they are collectively referred to as central hypothyroidism [3]. The diagnosis of hypothyroidism is based on the determination of serum FT4 and TSH levels, while the measurement of FT3 is generally unnecessary, as the majority of T3 in the body is generated via the peripheral conversion of T4 [4].

Hashimoto’s thyroiditis is a chronic autoimmune disorder of the thyroid gland that can progress from an asymptomatic euthyroid stage to overt hypothyroidism [5,6]. It is characterized by the presence of autoantibodies against thyroid peroxidase (anti-TPO) or thyroglobulin (anti-Tg), accompanied by the destruction of the glandular parenchyma, lymphocytic infiltration, and fibrotic tissue formation within the thyroid tissue itself [7].

Thyroid function is influenced by many external factors. Epidemiological studies demonstrate that current smokers have higher anti-TPO antibody titers, with the highest prevalence of positivity observed in individuals smoking more than 20 cigarettes per day [8].

Additionally, large population-based studies indicate a clear hereditary influence; first-degree relatives of affected patients have a 1.77-fold higher risk of developing the disease compared to the general population, along with a noticeable impact of shared environmental factors among spouses [9]. Furthermore, although clinical data remain limited, it is hypothesized that an elevated body mass index (BMI), specifically visceral adipose tissue, acts as an immunoregulatory organ. Through the secretion of adipokines and pro-inflammatory cytokines, it triggers low-grade chronic inflammation and disrupts the function of regulatory T-cells [10].

Recently, significant attention has been focused on researching the role of vitamin D as a potent immunomodulatory hormone that regulates both innate and adaptive immunity. Vitamin D is a fat-soluble vitamin, which exists in primary forms: the D2 form that can be found in certain plant-based foods, and the D3 form, which is present in an inactive state within human skin and is activated through direct exposure to sunlight [11]. By binding to specific vitamin D receptors (VDR) on immune cells, vitamin D suppresses excessive inflammation and autoimmune responses. Vitamin D deficiency has been associated with an increased risk of developing autoimmune thyroid diseases [12]. Conversely, recent meta-analyses have confirmed that vitamin D supplementation significantly reduces anti-TPO and anti-Tg antibody titers in patients with Hashimoto’s thyroiditis, while simultaneously improving thyroid function parameters [13].

In the context of external factors influencing the development of autoimmune thyroid disease, the season of birth has been investigated as an indirect ecological proxy for potential solar radiation exposure and maternal vitamin D status during intrauterine development, particularly during the first trimester. However, it should be noted that season of birth does not directly measure individual maternal ultraviolet exposure or serum vitamin D levels, as it can be confounded by factors such as maternal lifestyle, geographic residence, vitamin supplementation, and gestational age. While some studies investigating the influence of birth month on autoimmune thyroid diseases have failed to establish a clear association with individual months, broader seasonal groupings may reveal underlying patterns, thereby warranting a comparative analysis with patients suffering from non-autoimmune hypothyroidism [14,15].

Given the complex immunomodulatory role of vitamin D and the potential influence of early-life environmental factors, the primary objective of this study was to evaluate the association between season of birth and the development of Hashimoto’s thyroiditis compared to non-autoimmune hypothyroidism. As secondary objectives, the study aimed to compare serum vitamin D status, BMI, smoking status, family history of thyroid disease, and routine thyroid function parameters (TSH, fT4, fT3) between these two distinct hypothyroid patient groups, as well as to assess the potential association between serum vitamin D levels and BMI.

The study proposal and design for this research were previously presented in the Young Teachers Session at the XVII Congress of DNOOM (Clinical Excellence in Family Medicine - From Guidelines to Practice), held in Zagreb, Croatia, from March 5 to 8, 2026.

## Materials and methods

Study design and settings

This retrospective, observational, cross-sectional study was conducted at the Department of Nuclear Medicine and Radiology of a tertiary care university hospital in Bosnia and Herzegovina. The study included patients diagnosed with Hashimoto’s thyroiditis, as well as patients with non-autoimmune hypothyroidism, who were evaluated and followed up at this institution over a three-month period, from April 1 to June 30, 2026. Clinical, anthropometric, and laboratory data were retrieved from available medical records (medical history and electronic health charts) and analyzed retrospectively.

Participants

The study enrolled a total of 200 participants, divided into two groups: patients with non-autoimmune hypothyroidism (n = 100) and patients with Hashimoto’s thyroiditis (n = 100). Diagnoses were established based on clinical evaluation, thyroid function tests, thyroid ultrasound findings, and thyroid autoantibody status. Hashimoto’s thyroiditis was defined by the presence of serum anti-TPO and/or anti-Tg autoantibodies exceeding the laboratory reference threshold for positivity, alongside characteristic parenchymal hypoechogenicity on thyroid ultrasound. Non-autoimmune hypothyroidism was operationally defined as confirmed primary hypothyroidism (elevated TSH with low or normal fT4) with negative anti-TPO and anti-Tg antibodies (within the reference normal range) and the absence of ultrasonographic features characteristic of thyroid autoimmunity demographic, anthropometric, clinical, and biochemical variables, including age, sex, BMI, smoking status, family history of thyroid diseases, serum vitamin D levels, thyroid-stimulating hormone (TSH), free triiodothyronine (FT3), and free thyroxine (FT4) were retrospectively retrieved from available medical records and electronic health charts from routine outpatient evaluations.

Patients were included in the study if they had a confirmed diagnosis of either non-autoimmune hypothyroidism or Hashimoto’s thyroiditis, alongside complete medical records containing baseline demographic, anthropometric, and clinical data (age, sex, BMI, smoking status, disease duration, and family history). Additionally, available laboratory measurements for serum vitamin D, thyroid hormones (TSH, FT3, FT4), and thyroid autoantibodies (anti-TPO and anti-Tg) were required for inclusion.

Exclusion criteria comprised incomplete medical charts, a confirmed diagnosis of Graves' disease, a history of thyroid malignancy, prior thyroidectomy, radioiodine therapy, drug-induced thyroid dysfunction, or central (secondary or tertiary) hypothyroidism. Patients who were pregnant at the time of evaluation or taking medications known to affect thyroid function - other than standard thyroid hormone replacement therapy - were also excluded from the study.

Data on body weight and height were obtained from available medical records, where they were originally measured and recorded during clinical evaluation according to standardized procedures. BMI was calculated as body weight in kilograms divided by the square of height in meters (kg/m²). BMI was categorized into four standard groups: underweight (< 18.5 kg/m²), normal weight (18.5-24.9 kg/m²), overweight (25.0-29.9 kg/m²), and obese (≥ 30.0 kg/m²). Season of birth was defined based on the month of birth as spring (March-May), summer (June-August), autumn (September-November), or winter (December-February). Smoking status was categorized based on medical record entries as current active smokers versus non-smokers. A positive family history of thyroid diseases was defined as any recorded diagnosis of thyroid dysfunction or thyroid disease among close family members. Thyroid function was assessed based on serum levels of TSH, FT3, and FT4, while thyroid autoimmunity was evaluated via anti-TPO and anti-Tg antibody levels. Furthermore, serum TSH levels were categorized according to the laboratory reference range as low (< 0.34 mIU/L), normal (0.34-4.95 mIU/L), or elevated (> 4.95 mIU/L). Vitamin D status was assessed based on its serum concentration.

Statistical analysis

Statistical data analysis was performed using the IBM SPSS Statistics software, version 26.0 (IBM Corp., Armonk, NY). The normality of continuous variables was evaluated using the Shapiro-Wilk and Kolmogorov-Smirnov tests. Since the continuous data (including age, BMI, serum vitamin D, and thyroid hormone levels) deviated from a normal distribution, they were expressed as medians with interquartile ranges (IQR) and minimum-maximum (min-max) ranges. Categorical variables (such as sex, smoking status, family history of thyroid disease, and season of birth) were presented as absolute frequencies and percentages (n, %). Differences in continuous variables between the two study groups (Hashimoto’s thyroiditis vs. non-autoimmune hypothyroidism) were evaluated using the non-parametric Mann-Whitney U test. For comparisons of continuous variables across three or more categories (e.g., vitamin D levels across different BMI categories), the Kruskal-Wallis H test was applied. Post hoc pairwise comparisons for significant Kruskal-Wallis findings were conducted using the Bonferroni correction to adjust for multiple testing. Differences in categorical variables between the investigated groups were analyzed using the chi-square (χ²) test of independence. A two-tailed p-value of less than 0.05 was considered statistically significant.

Ethical considerations

The protocol of this study was approved by the Ethics Committee of the University Clinical Center Tuzla (Tuzla, Bosnia and Herzegovina) under protocol number 02-09/2-110-2/26, dated June 29, 2026. The research was conducted in strict accordance with the ethical principles of the Declaration of Helsinki for medical research involving human subjects. Owing to its retrospective design and the use of anonymized patient data, the requirement for written informed consent was waived by the ethics committee.

## Results

The study included a total of 200 participants, equally divided into two groups of 100. Females comprised the vast majority of the overall study population, with 189 (94.5%) females and 11 (5.5%) males. Specifically, there were 95 (95.0%) females in the Hashimoto’s thyroiditis group and 94 (94.0%) females in the non-autoimmune hypothyroidism group. There was no statistically significant difference in sex distribution between patients with hypothyroidism and patients with Hashimoto’s thyroiditis (χ² = 0.096, df = 1, p = 0.756) (Table 1).

**Table 1:** Sex distribution of patients in the investigated patient groups n - number of patients Data are presented as n (%). Group comparison was performed using Pearson's chi-square test.

Variable	Hypothyroidism n (%)	Hashimoto's thyroiditis n (%)	χ²	df	p-value
Male	6 (6.0)	5 (5.0)	0.096	1	0.756
Female	94 (94.0)	95 (95.0)	-	-	-

Patients with non-autoimmune hypothyroidism were significantly older than patients with Hashimoto’s thyroiditis, with a median age of 59.0 years compared with 49.0 years, respectively (Mann-Whitney U = 3386.500, Z = -3.944, p < 0.001). Patients with non-autoimmune hypothyroidism had significantly higher BMI values compared with patients with Hashimoto’s thyroiditis, with median BMI values of 28.20 kg/m² and 26.21 kg/m², respectively (Mann-Whitney U = 3913.500, Z = -2.655, p = 0.008). There was no statistically significant difference in vitamin D levels between patients with hypothyroidism and patients with Hashimoto’s thyroiditis, with median values of 20.75 and 21.70, respectively (Mann-Whitney U = 4964.500, Z = -0.087, p = 0.931). There was no statistically significant difference in TSH levels between patients with hypothyroidism and patients with Hashimoto’s thyroiditis, with median TSH values of 2.37 mIU/L and 2.62 mIU/L, respectively (Mann-Whitney U = 4524.000, Z = -1.163, p = 0.245). There was no statistically significant difference in fT3 levels between patients with hypothyroidism and patients with Hashimoto’s thyroiditis, with median fT3 values of 4.49 and 4.42, respectively (Mann-Whitney U = 4646.000, Z = -0.748, p = 0.454). There was no statistically significant difference in fT4 levels between patients with non-autoimmune hypothyroidism and patients with Hashimoto’s thyroiditis, with median fT4 values of 16.80 pmol/L and 16.10 pmol/L, respectively (Mann-Whitney U = 4547.500, Z = -1.106, p = 0.269) (Table 2).

**Table 2:** Baseline characteristics of patients in the investigated patient groups BMI - body mass index; fT3 - free triiodothyronine; fT4 - free thyroxine; IQR - interquartile range; TSH - thyroid-stimulating hormone Continuous variables are presented as minimum-maximum, median, and interquartile range (IQR). Group comparisons were performed using the Mann-Whitney U test. *Statistically significant at p < 0.05.

Variable	Hypothyroidism min-max	Median	IQR	Hashimoto's thyroiditis min-max	Median	IQR	Mann-Whitney U	Z	p-value
Age, years	17.00-87.00	59.00	17.00	21.00-79.00	49.00	20.00	3386.500	-3.944	<0.001
BMI, kg/m²	18.47-48.22	28.20	7.60	17.30-36.85	26.21	5.81	3913.500	-2.655	0.008
Vitamin D, nmol/L	5.00-211.00	20.75	12.00	6.00-66.00	21.70	11.00	4964.500	-0.087	0.931
TSH, mIU/L	0.01-41.03	2.37	2.78	0.02-19.79	2.62	3.68	4524.000	-1.163	0.245
fT3, pmol/L	2.37-13.60	4.49	1.05	2.72-6.08	4.42	0.89	4646.000	-0.748	0.454
fT4, pmol/L	6.35-29.98	16.80	5.30	6.96-25.01	16.10	4.51	4547.500	-1.106	0.269

The distribution of birth season did not differ significantly between patients with non-autoimmune hypothyroidism and patients with Hashimoto’s thyroiditis (χ² = 2.444, df = 3, p = 0.485). In the hypothyroidism group, the highest proportion of patients was born in winter (30.0%), followed by autumn (26.0%), summer (23.0%), and spring (21.0%). In the Hashimoto’s thyroiditis group, the highest proportions were observed for summer and autumn births (29.0% each), followed by spring and winter births (21.0% each) (Table 3).

**Table 3:** Distribution of birth season in the investigated patient groups n - number of patients Data are presented as n (%). Group comparison was performed using Pearson's chi-square test.

Birth season	Hypothyroidism n (%)	Hashimoto's thyroiditis n (%)	χ²	df	p-value
Winter	30 (30.0)	21 (21.0)	2.444	3	0.485
Spring	21 (21.0)	21 (21.0)	-	-	-
Summer	23 (23.0)	29 (29.0)	-	-	-
Autumn	26 (26.0)	29 (29.0)	-	-	-
Total	100 (100.0)	100 (100.0)	-	-	-

There was no statistically significant difference in smoking status between patients with hypothyroidism and patients with Hashimoto’s thyroiditis (22.0% vs. 23.0%, respectively; χ² = 0.029, df = 1, p = 0.866) (Table 4).

**Table 4:** Smoking status in the investigated patient groups n - number of patients Data are presented as n (%). Group comparison was performed using Pearson's chi-square test.

Smoking status	Hypothyroidism n (%)	Hashimoto's thyroiditis n (%)	χ²	df	p-value
No	78 (78.0)	77 (77.0)	0.029	1	0.866
Yes	22 (22.0)	23 (23.0)	-	-	-

In the non-autoimmune hypothyroidism group, vitamin D levels differed significantly across BMI categories (Kruskal-Wallis H = 8.940, df = 3, p = 0.030). The lowest median vitamin D level was observed in overweight patients. In contrast, no statistically significant difference in vitamin D levels across BMI categories was observed in the Hashimoto’s thyroiditis group (Kruskal-Wallis H = 2.365, df = 3, p = 0.500) (Table 5).

**Table 5:** Vitamin D levels according to BMI category in the investigated patient groups BMI - body mass index; IQR - interquartile range; n - number of patients Vitamin D values are presented as minimum-maximum, median, and IQR. Comparisons among BMI categories were performed using the Kruskal-Wallis test. *Statistically significant at p < 0.05.

Group	BMI category	n	Min-max	Median	IQR	Kruskal-Wallis H	df	p-value
Hypothyroidism	Underweight	1	-	-	-	8.940	3	0.030*
	Normal weight	21	5-64	24.00	19			
	Overweight	41	10-45	18.50	9			
	Obesity	37	10-211	23.00	13			
Hashimoto's thyroiditis	Underweight	1	-	-	-	2.365	3	0.500
	Normal weight	33	10-43	25.50	16			
	Overweight	44	6-65	20.35	8			
	Obesity	22	13-66	22.30	10			

Post hoc pairwise comparisons with Bonferroni correction showed that, within the non-autoimmune hypothyroidism group, overweight patients had significantly lower vitamin D levels compared with patients with normal body weight (median 18.50 vs. 24.00, adjusted p = 0.045). No other pairwise comparisons remained statistically significant after adjustment.

A positive family history was significantly more frequent among patients with Hashimoto’s thyroiditis compared with patients with hypothyroidism (62.0% vs. 47.0%, respectively; χ² = 4.537, df = 1, p = 0.033) (Table 6).

**Table 6:** Family history of thyroid diseases in the investigated patient groups n - number of patients Data are presented as n (%). Group comparison was performed using Pearson's chi-square test. *Statistically significant at p < 0.05.

Family history	Hypothyroidism n (%)	Hashimoto's thyroiditis n (%)	χ²	df	p
Negative	53 (53.0)	38 (38.0)	4.537	1	0.033*
Positive	47 (47.0)	62 (62.0)	-	-	-

## Discussion

In this study, we evaluated demographic, clinical, and laboratory characteristics to distinguish Hashimoto’s thyroiditis from non-autoimmune hypothyroidism, identifying key differences in BMI and family history.

The majority of our study population consisted of female patients, with a nearly identical percentage distribution observed between the two groups. This female predominance is highly consistent with global epidemiological data. For instance, a large-scale meta-analysis comprising 48 studies demonstrated that the prevalence of Hashimoto’s thyroiditis is substantially higher in females, reaching up to 17.5%, compared to only 6.0% in males [16]. Since the non-autoimmune hypothyroidism also predominantly affects females, it can be concluded that the sex distribution of our patients aligns well with well-established epidemiological data [17].

In a study conducted by a group of authors, BMI was compared between females with Hashimoto’s thyroiditis and those who reported being healthy. Their results demonstrated that females with Hashimoto’s thyroiditis were more likely to have a body weight deviating from the normal range compared to their healthy control group. While our study compared patients being treated for Hashimoto’s thyroiditis with those treated for non-autoimmune hypothyroidism, our findings also reveal an overall median BMI greater than 25 kg/m² in both investigated groups, further suggesting a notable deviation from normal body weight [18].

However, our study revealed a statistically significant difference in BMI values between these two pathologies, with notably higher values observed in the non-autoimmune hypothyroidism group compared to the Hashimoto’s thyroiditis group (median: 28 kg/m², versus 26 kg/m²). Furthermore, serum vitamin D levels in our study did not demonstrate statistical significance when compared between the two investigated groups. These findings correlate well with the study conducted in Iran, which yielded similar results. Specifically, those authors also found no statistically significant difference in vitamin D levels between patients with Hashimoto’s thyroiditis compared to those with non-autoimmune hypothyroidism. However, as expected, they demonstrated that both groups had significantly lower serum vitamin D levels compared to their healthy control group [19].

However, given the substantial age difference between our study cohorts (median: 59.0 vs. 49.0 years), the observed disparity in BMI cannot be attributed independently to the disease category alone without adjusted multivariable modeling.

When comparing our findings with the previously mentioned study by Hamilton et al. [14], which investigated the influence of birth month on the development of autoimmune thyroid disease in the United Kingdom, we observed that grouping patients by birth season similarly failed to reach statistical significance. However, minor variations can be noted. In the United Kingdom study, females with Hashimoto’s thyroiditis were more frequently born during the autumn and winter months. In contrast, in our study, patients with Hashimoto’s thyroiditis were equally distributed between summer and autumn births (29 patients each), while 21 patients each were born during the winter and spring seasons.

On a broader regional level, seasonal patterns of Hashimoto’s thyroiditis have also been explored using alternative methodologies. Marcec et al. [15] analyzed 17-year Google Trends search data and found no significant seasonality in search interest for Bosnia and Herzegovina, unlike in neighboring countries (Serbia, Montenegro, Croatia). Although online search activity reflects clinical awareness rather than season of birth, these data provide additional regional context regarding temporal patterns in thyroid autoimmunity.

Given the limited sample size in our study, we cannot definitively rule out the possibility that prenatal solar radiation influences the subsequent development of Hashimoto’s thyroiditis. Additionally, the lack of complete data regarding the gestational age at birth represents a further limitation of our study.

Among other environmental factors influencing the pathogenesis and clinical course of Hashimoto’s thyroiditis, cigarette smoking is frequently highlighted in the literature, with some studies pointing toward an unexpected protective effect. Specifically, several investigations have demonstrated that serum thyroid autoantibody levels are significantly lower in active smokers compared to non-smokers [20].

Conversely, a large-scale study involving euthyroid subjects that investigated the effects of cigarette smoking on TSH, T3, T4, anti-TPO, and anti-Tg levels actually demonstrated that the lower TSH values were recorded in smokers, whereas anti-TPO levels were significantly higher in individuals smoking more than 20 cigarettes per day [8]. These discrepancies suggest that the impact of tobacco smoke on thyroid function and autoimmune processes may be complex and dose-dependent, particularly when observed within a healthy population.

The findings of our study showed no statistically significant difference in the frequency of cigarette smoking between the investigated groups. However, it should be noted that in our study, thyroid hormone and autoantibody levels were not directly correlated with the patients' smoking status, which leaves an open avenue for future, more detailed investigations within our population.

This study has several limitations. The retrospective design limits causal interpretation, and all clinical and laboratory data, including serum vitamin D levels, were extracted from medical records without individual tracking of the seasonal timing of blood collection or vitamin D supplementation status. Furthermore, a statistically significant age difference was observed between the two study groups, which represents a potential confounding factor when comparing BMI, family history, and other clinical characteristics. The sample size was also modest, particularly in subgroup analyses such as male patients, low TSH, and underweight BMI categories. Treatment-related variables, including levothyroxine dose and duration, were not incorporated into the analysis, although recent evidence suggests that levothyroxine dose alone may not fully explain antibody levels. Additional factors, such as iodine intake, selenium status, waist circumference, lipid profile, insulin resistance, inflammatory markers, thyroid ultrasound features, and smoking intensity, were also not assessed. Finally, because this was a single-center study, the findings may not be fully generalizable to broader populations.

## Conclusions

In conclusion, this study demonstrates that birth season, smoking status, and overall serum vitamin D levels do not significantly differ between patients with Hashimoto’s thyroiditis and those with non-autoimmune hypothyroidism. However, patients with non-autoimmune hypothyroidism exhibited a higher median BMI, and a lower vitamin D level was more frequently observed among overweight individuals in this group. Conversely, Hashimoto’s thyroiditis was associated with a higher frequency of reported family history of thyroid disease. These findings highlight differences in the clinical and epidemiological profiles of these conditions, while acknowledging that observed associations, particularly regarding BMI and family history, may be influenced by age disparities and other unadjusted confounders. Future prospective studies are needed to further clarify environmental and hereditary factors in thyroid disease.
